# Kinetin Capped Zinc Oxide Nanoparticles Improve Plant Growth and Ameliorate Resistivity to Polyethylene Glycol (PEG)-Induced Drought Stress in *Vigna radiata* (L.) R. Wilczek (Mung Bean)

**DOI:** 10.3390/molecules28135059

**Published:** 2023-06-28

**Authors:** Maham Ajmal, Rehman Ullah, Zahir Muhammad, Muhammad Nauman Khan, Hussain Ahmad Kakar, Alevcan Kaplan, Mohammad K. Okla, Ibrahim A. Saleh, Asif Kamal, Abdullah Abdullah, Sarah Abdul Razak

**Affiliations:** 1 Department of Botany, University of Peshawar, Peshawar 25120, Pakistan; m.hy@gmail.com (M.A.); zahid.umar@gmail.com (Z.M.); hu.ahmed@gmail.com (H.A.K.); 2 Department of Botany, Islamia College Peshawar, Peshawar 25120, Pakistan; 3 University Public School, University of Peshawar, Peshawar 25120, Pakistan; 4 Department of Crop and Animal Production, Sason Vocational School, Batman University, Batman 72060, Turkey; kaplanalevcan@gmail.com; 5 Botany and Microbiology Department, College of Science, King Saud University, P.O. Box 2455, Riyadh 11451, Saudi Arabia; malokla@ksu.edu.sa; 6 Faculty of Science, Zarqa University, Zarqa 13110, Jordan; isaleh@zu.edu.jo; 7 Department of Plant Sciences, Faculty of Biological Sciences, Quaid-i-Azam University, Islamabad 45320, Pakistan; 8 Faculty of Biology, University of Munich (LMU), 82152 Munich, Germany; 9 Institute of Biological Sciences, Faculty of Science, Universiti Malaya, Kuala Lumpur 50603, Malaysia

**Keywords:** antioxidant enzymes, chlorophyll contents, Kn-ZnONPs, mung bean, PEG-induced drought stress

## Abstract

Plants are sessile and mostly exposed to various environmental stresses which hamper plant growth, development, and significantly decline its production. Drought stress is considered to be one of the most significant limiting factors for crop plants, notably in arid and semi-arid parts the world. Therefore, the present study aimed to evaluate the potential impact of different concentrations (10, 100, and 200 µg/mL) of kinetin capped zinc oxide nanoparticles (Kn-ZnONPs) on *Vigna radiata* (L.) R. Wilczek under varying levels (5%, 10%, 15%) of PEG-induced drought stress. ZnONPs were synthesized by a co-precipitation method using Zinc acetate as a precursor at pH-12, incinerated to 500 °C, and kinetin was used as a surface functionalizing agent. The resulting Kn-ZnONPs were characterized by various contemporary analytical techniques, including SEM, SEM-EDS, XRD, DLS, and Zeta potential and IR spectroscopy. Crystalline Kn-ZnONPs, with a zeta potential of 27.8 mV and a size of 67.78 nm, of hexagonal wurtzite structure and vibrational stretches associated with N-H, C-O, C-N, etc., were confirmed. PEG-induced drought stress significantly reduced the growth of *V. radiata* by declining the chlorophyll and carotenoid contents. Moreover, a significant decrease in the levels of superoxide dismutase (SOD), peroxidase (POD), catalase (CAT), ascorbate peroxidase (APX), soluble sugar contents, proline, protein contents, phenol, and tannin were observed compared to the control. However, the exogenous application of Kn-ZnONPs ameliorated all photosynthetic parameters by up-regulating the antioxidant defense system through the promotion of SOD, POD, CAT, and lipid peroxidation levels. The biochemical parameters, such as proteins, soluble sugars, and proline, were observed to be maximum in plants treated with 200 µg/mL Kn-ZnONPs under 5% drought stress. The application of Kn-ZnONPs also enhanced the total phenol contents, flavonoid, and tannin contents. In conclusion, the findings of this study demonstrate that the exogenous application of Kn-ZnONPs provides beneficial effects to *V. radiata* by attenuating the damaging effects of drought stress through the up-regulation of the antioxidant defense system and osmolytes. These results suggest that Kn-ZnONPs have potential as a novel approach to improve crop productivity under drought stress conditions.

## 1. Introduction

Drought is a major stress factor that significantly impacts agricultural productivity, leading to severe economic losses and food insecurity worldwide. According to the Food and Agriculture Organization (FAO), droughts are responsible for over 80% of crop losses in developing countries, resulting in an estimated annual global economic loss of over 6 billion USD [1]. The available literature highlights the significant impact of drought on agricultural productivity and the associated economic losses. Mishra et al. [2] showed that drought has a significant negative impact on crop yields in several countries, including Pakistan. Baloch et al. [3] highlighted the negative impact of drought on wheat productivity in Pakistan, with yield reductions of up to 50% reported during drought periods. Pakistan, being an agrarian economy, is particularly vulnerable to the impact of drought on agriculture. The country has experienced several droughts in the past, with the most recent being the severe drought of 2018–2019, which led to a decline in crop yields and agricultural productivity, affecting the livelihoods of millions of people [4]. Drought is a significant challenge for Pakistan’s agriculture sector, which is already facing several other challenges such as water scarcity, low productivity, and climate change impacts [5]. Agriculture contributes around 18.9% to Pakistan’s GDP and provides employment to over 40% of its labor force [6]. Droughts in Pakistan result in lower crop yields and a decrease in livestock productivity, affecting the country’s exports of agricultural products and increasing food insecurity [7]. Estimates suggest that droughts cost Pakistan around 1.8 billion USD annually [8]. Environmental changes drastically affect the natural system, human health, and agricultural productivity, especially in the developing world [9]. Biotic and abiotic stresses caused by environmental variations have deleterious effects on the agriculture of a region. Environmental changes affecting the lands and agriculture include the rise in average temperature, changes in the pattern of annual rainfall, heat waves, global variation in atmospheric carbon dioxide level, and fluctuation in sea level [10]. Drought stress poses a significant threat to the process of germination and the growth of seedlings. It negatively impacts the levels of photosynthetic pigments, the functionality of membranes, and the activity of enzymes, ultimately leading to a considerable decline in crop yield. Specifically, the vegetative phase and the initial flowering stage of development are particularly vulnerable to these adverse effects [11,12]. The accumulation of ROS in plant parts leads to osmotic stress, negatively affecting the cellular transport system and causing porosity in cell membrane structure due to lipid peroxidation. Drought stress impairs plastid structure and negatively affects the photosynthetic system of plants [13].

Nanotechnology has the potential to mitigate environmental changes’ deleterious effect on crop production and play a crucial role in promoting the agricultural industry [14]. Nanoparticles, fabricated at a size of 1 to 100 nm, have unique and inspiring properties due to their small size and large surface-to-volume ratios [15,16]. Nanoparticle-based plant transformation technology offers a more efficient way to genetically modify plants compared to traditional methods. Nanoparticles, due to their small size, effectively transport foreign substances into plant cells while safeguarding them from degradation. Moreover, nanoparticles provide a novel approach for crop protection against specific agricultural issues [17,18]. Plants can uptake nanoparticles through various pathways, such as stomata, root hairs, and leaf surface cracks. Once inside the plant, nanoparticles can move through diffusion, bulk flow, and phloem loading. Several factors, including particle size, shape, surface properties, solution pH, and the presence of other substances, influence the transport of nanoparticles. Previous studies have employed different application methods, such as leaf spraying, root application, branch injection, and seed treatment, confirming the uptake of nanoparticles by plants [18,19]. Several types of nanoparticles, including silver, gold, platinum, titanium, cerium oxide, and zinc oxide nanoparticles, have numerous applications in different fields, including agriculture [20,21]. Among these nanoparticles, zinc oxide (ZnONPs) is of great interest due to its wide range of applications and has been utilized in various fields of biological science and agriculture [22]. ZnONPs are reported to be the most exploit metallic nanoparticles as they have numerous applications in the semiconductor industry, agriculture, and the biomedical field [21]. The antibacterial potential, antifungal efficacy, catalytic, UV-filtering properties, and role in pharmaceutical and cosmetic industries of ZnONPs are also well documented [23,24,25]. However, the use of kinetin-caped zinc oxide nanoparticles (Kn-ZnONPs) has not been reported previously. Kinetin is considered the most important cytokines which has the potential to improve germination, plant growth, and various physio-chemical process occurring in the plant via a signal transduction system mitigate cell division and regulate the functions of those enzymes which transport sugar and reduce nitrates [26,27]. Kinetin also promotes embryogenesis and improves the meristems of new growing roots and shoots, enhances nodule formation, and increases the number of young roots [28].

The current study is aimed at the application of Kn-ZnONPs as a stress mitigation agent and seeks to investigate the potential application of Kn-ZnONPs in ameliorating the tolerance level of mung beans to PEG-based drought stress.

## 2. Results

### 2.1. Characterization of Kn-ZnONPs

**Figure 1:**
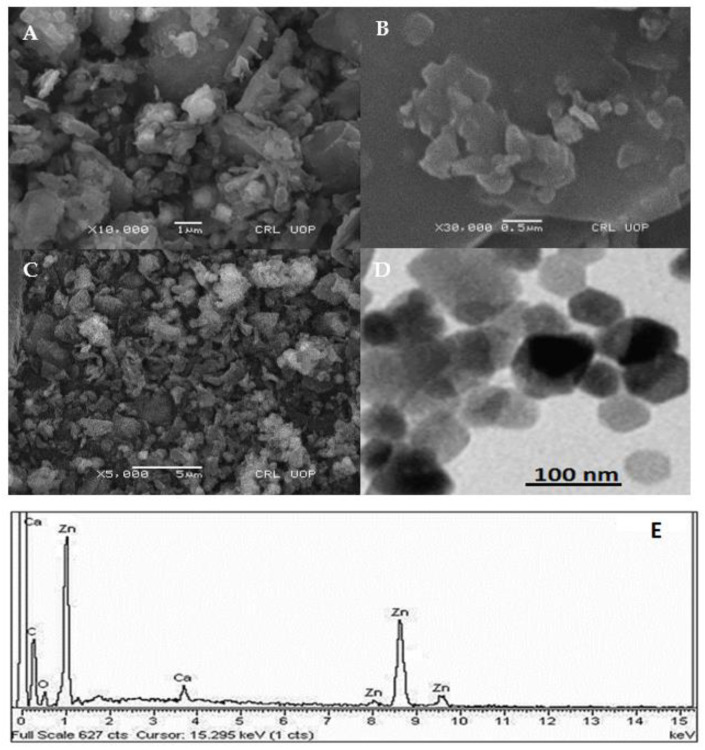
Scanning electron microscopy (SEM) images (**A**–**C**) at various magnification and spatial resolution of 0.5 µm to 5 µm, Transmission electron Micrograph (TEM) of kinetin capped ZnONPs (**D**) and SEM-EDS showing elemental Zinc signals as core of Zinc-oxide nanoparticles at different keV (**E**).

**Figure 2:**
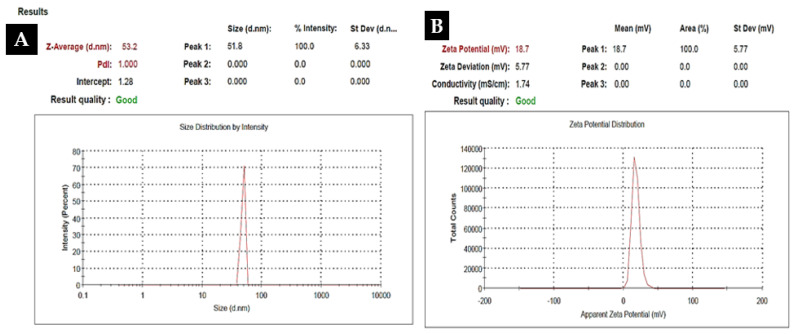
Dynamic light scattered spectrogram (DLS) of Kn-ZnONPs showing mean hydrodynamic particles size of 53.2 nm (**A**) with a Zeta potential of 18.7 mV (**B**).

**Figure 3:**
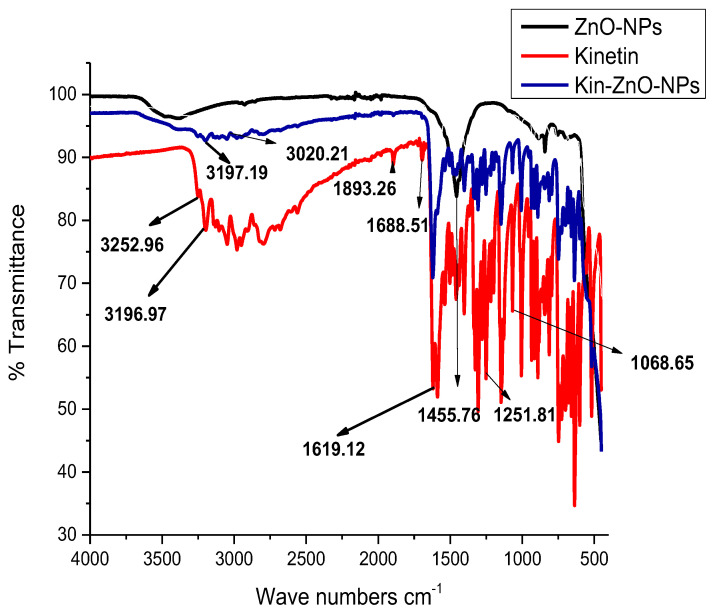
Fourier Transform Infrared (FT-IR) Spectroscopy of Kinetin, ZnONPs, and Kn-ZnONPs showing different vibration stretches at various wavenumbers cm^−^^1^ corresponding to the groups associated with kinetin and helped in the binding/capping and stabilization of ZnONPs suspension.

**Figure 4:**
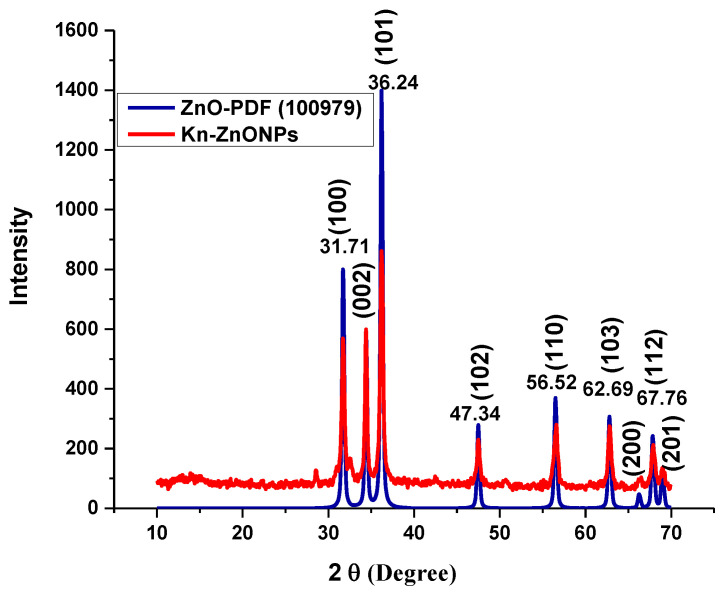
X-ray powder diffractogram of Kn-ZnONPs (red) matching the standard ZnO diffractogram (blue) of PDF-100979, showing various intensities at different 2 Ɵ levels, correspond to various Bragg’s planes of hexagonal wurtzite lattice.

To investigate the morphology, size and size distribution, surface charge, crystalline structure, and functional groups involved in the synthesis of Kn-ZnONPs, a contemporary technique was utilized. SEM and TEM analysis of Kn-ZnONPs showed polydispersion in size, ranging from 30–92 nm with spherical morphology (Figure 1A–D). The EDX analysis of Kn-ZnONPs elucidated the signals of elemental zinc at 2.1 keV due to the SPR band, validating the existence of core zinc in Kn-ZnONPs (Figure 1E). DLS spectrogram revealed a unimodel size distribution with a mean hydrodynamic size of 53.2 nm and a zeta potential of 18.7 mV (Figure 2). The FT-IR spectra of Kn-ZnONPs showed the presence of functional groups such as C=C stretching (α, β unsaturated ketone), N-H stretching (secondary amine), C-H stretching (alkane, methyl group), strong O-H stretching, N-C bending (amine), N=C stretching (oxime), C-H bending (aromatic), and C-O stretching (aromatic ester) depicted characteristic bands at 1619.12 cm^−1^, 3252.96 cm^−1^, 3197.19 cm^−1^, 3020.21 cm^−1^, 1068.65 cm^−1^, 1640–16901cm^−1^, 1893.26 cm^−1^, and 1251.81 cm^−1^, respectively, confirming the capping of kinetin on core zinc oxide nanoparticles (Figure 3). The crystalline structure of Kn-ZnONPs was determined via X-ray diffraction (XRD) technique. The analysis of Kn-ZnONPs showed characteristic peaks at 31.71°, 36.24°, 47.34°, 56.52°, 62.69°, and 67.76°, correspond to Bragg’s planes of (100), (101), (102), (110), (103), and (200) (Figure 4), and confirming hexagonal wurtzite lattice geometry. The Scherrer’s equation was applied to calculate the average size (24.27 nm) of Kn-ZnONPs by determining the full width at half maximum (FWHM) of the (101) Bragg’s reflection.

### 2.2. Effect of Kn-ZnONPs on Seed Germination and Agronomic Profile

**Figure 5:**
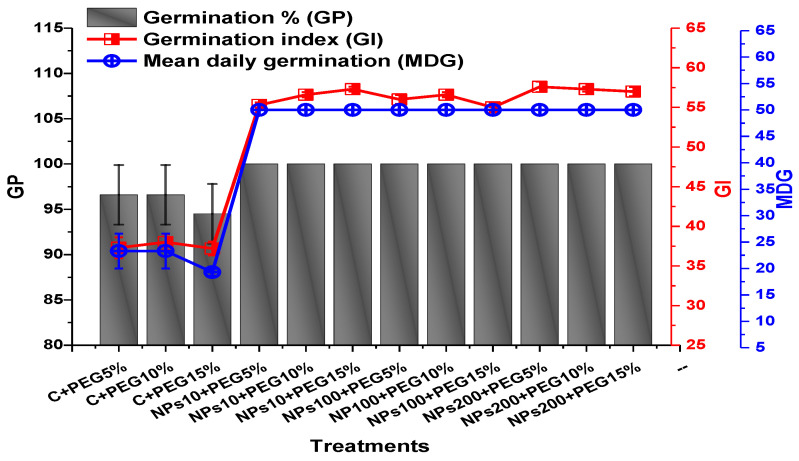
Showing the impact of priming of various doses of Kn-ZnONPs on various germination indices (GP, GI, and MDG) of *V. radiata* grown under different levels of PEG-induced drought stress.

**Figure 6:**
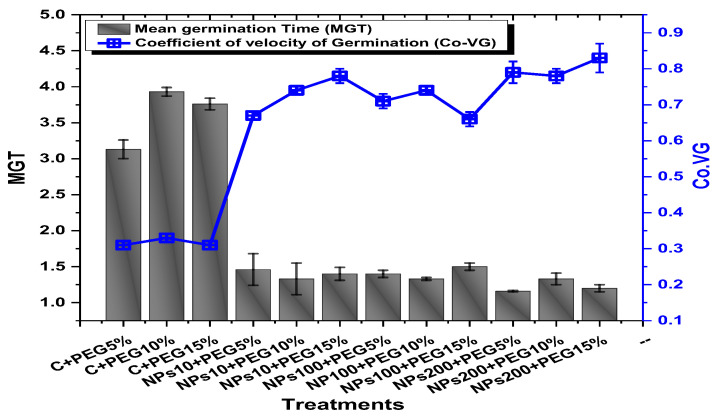
Showing the impact of priming of various doses of Kn-ZnONPs on various germination indices (MGT and Co-VG) of *V. radiata* grown under different levels of PEG-induced drought stress.

The application of PEG-induced drought significantly decreased the germination percentage (94.5%), germination index (37.2), mean daily germination (19.3), and coefficient of velocity germination (0.31) of mung beans, while the application of Kn-ZnONPs mitigated the adverse effect of PEG-induced drought stress (Figure 5 and Figure 6). The mean germination duration decreased in seeds treated with different concentrations of Kn-ZnONPs. The effect of different concentrations of Kn-ZnONPs on germination percentage and mean daily germination (MDG) was the same (100% and 50%, respectively), while the mean germination time (MGT), germination index (GI), and coefficient of velocity germination (CVG) (1.16, 57.6, and 79, respectively) were dose-dependent, as the seeds treated with the maximum concentration of Kn-ZnONPs (200 µg/mL) had the minimum MGT, while the GI and CVG values increased with increasing the concentration of Kn-ZnONPs under different levels of PEG-based drought stress (Figure 5 and Figure 6).

**Figure 7:**
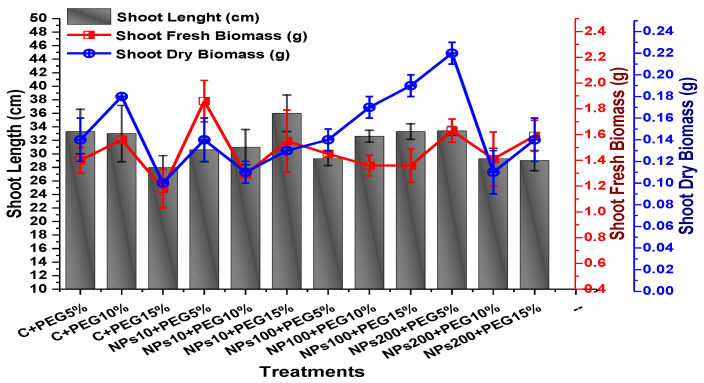
Showing the effect of priming and foliar application of various doses of Kn-ZnONPs on shoot length (cm) and fresh and dry biomasses (g) of *V. radiata* grown under different levels of PEG-induced drought stress.

**Figure 8:**
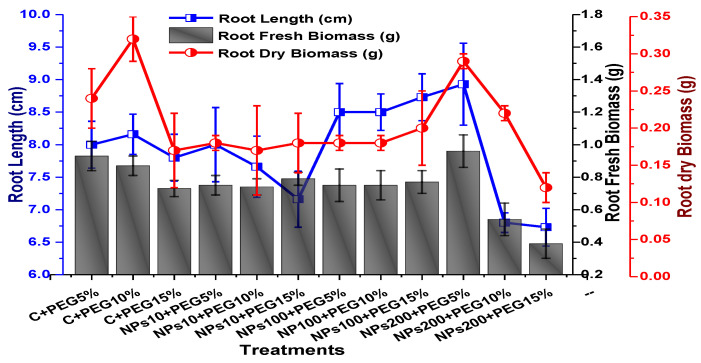
Showing the effect of priming and foliar application of various doses of Kn-ZnONPs on root length (cm) and fresh and dry biomasses (g) of *V. radiata* grown under different levels of PEG-induced drought stress.

**Figure 9:**
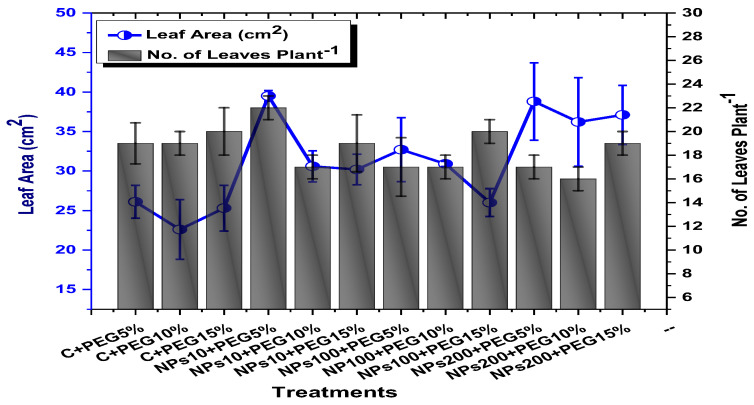
Showing the effect of priming and foliar application of various doses of Kn-ZnONPs on number of leaves per plant and leaf area (cm^2^) of *V. radiata* grown under different levels of PEG-induced drought stress.

The effects of Kn-ZnONPs on vegetative performance of *V. radiata* under varying levels of PEG-induced drought stress was analyzed by measuring shoot and root lengths and shoot and root fresh/dry biomasses. The results showed that foliar application with higher concentrations of Kn-ZnONPs generally resulted in better growth compared to the plants without Kn-ZnONPs. Specifically, the treatment with Kn-ZnONPs at 10 µg/mL had the highest shoot length (36 cm) under 15% PEG-based drought stress, while plants exposed to 15% PEG stress alone had the lowest (28 cm) shoot length. Moreover, the maximum mean shoot fresh biomass of 1.86 g was obtained from the plants treated with 100 µg/mL of Kn-ZnONPs and 5% PEG, while the lowest (1.18 g) was recorded in plants under 15% PEG-based stress. Foliar application of plants with 10µg/mL of Kn-ZnONPs at 15% PEG stress had the highest shoot dry mass (0.22 g), while the lowest (0.1 g) occurred in plants without Kn-ZnONPs under 15% PEG stress (Figure 7). For root length, the highest value (8.93 cm) was obtained from plants under 15% PEG drought stress treated with 100 µg/mL of foliar spray of Kn-ZnONPs, while the lowest (6.73 cm) was observed with 10 µg/mL of Kn-ZnONPs and 10% PEG stress. Similarly, the lowest (0.39 g) root fresh biomass was recorded in plants under 10% PEG stress treated with 10 µg/mL of Kn-ZnONPs, while the maximum (0.96 g) root biomass was obtained from the treatment with Kn-ZnONPs 200 µg/mL at 10% PEG stress (Figure 8). Leaf area was drastically affected by drought stress and the leaf area was calculated at a minimum (22.6 cm^2^) in plants facing 15% drought stress, while the maximum leaf area (39.5 cm^2^) was observed in this case in plants having treatments of 10 µg/mL Kn-ZnONPs and 5% drought stress (Figure 9). Taken together, these results show that the application of Kn-ZnONPs shows a synergistic effect on mung bean growth and extenuates the effect of drought stress.

### 2.3. Effect of Kn-ZnONPs on Photosynthetic Pigments and Osmoprotectants Assimilates

**Figure 10:**
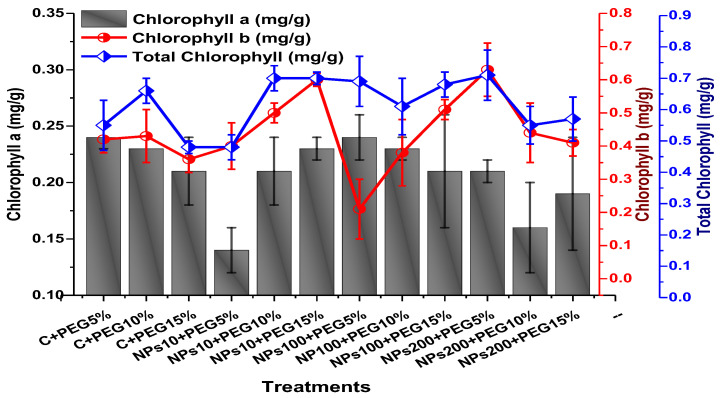
Showing the effect of priming and foliar application of various doses of Kn-ZnONPs on total chlorophyll and chlorophyll a and b (mg/g FW) of *V. radiata* grown under different levels of PEG-induced drought stress.

**Figure 11:**
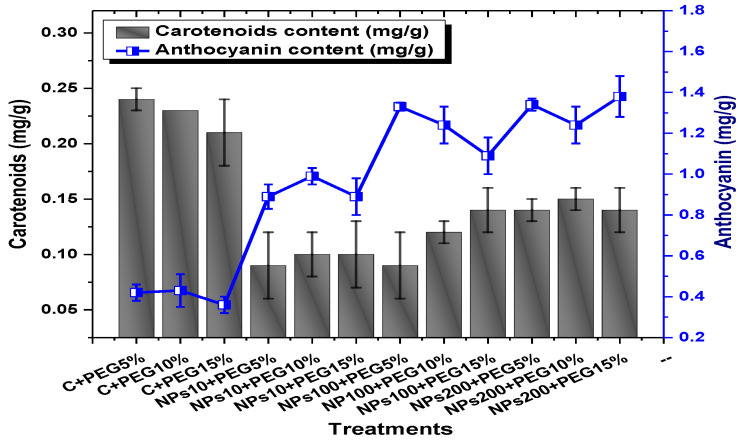
Showing the effect of priming and foliar application of various doses of Kn-ZnONPs on carotenoids and anthocyanin contents (mg/g FW) of *V. radiata* grown under different levels of PEG-induced drought stress.

The impact of foliar application of Kn-ZnONPs on the photosynthetic pigments included chlorophyll ‘a’, chlorophyll ‘b’, and carotenoids contents; total chlorophyll and anthocyanin contents were assessed in plants grown under varying levels of PEG-based drought stress. The results revealed that the photosynthetic pigments were significantly elevated by the application of Kn-ZnONPs when used in 200 µg/mL concentration under stress as compared to plants without applied Kn-ZnONPs (Figure 10 and Figure 11). The highest chlorophyll a content (0.28 mg/g) was observed in plants treated with Kn-ZnONPs 10 µg/mL at 5% PEG drought stress. Plants treated with Kn-ZnONPs 10 µg/mL at 10% PEG stress exhibited a chlorophyll a content of 0.23 mg/g, while plants treated with Kn-ZnONPs 10 µg/mL and PEG 15% exhibited a chlorophyll a content of 0.24 mg/g. Furthermore, plants at 5% PEG-based drought stress treated with 100 µg/mL Kn-ZnONPs and NPs 100 µg/mL and PEG 15%, exhibited a chlorophyll a content of 0.26 mg/g. However, plants treated with NPs 100 µg/mL and grown 10% PEG exhibited a chlorophyll a content of 0.21 mg/g. Similarly, the chlorophyll b content varied significantly among various treatments used (Figure 10 and Figure 11).

Among the different concentrations of Kn-ZnONPs tested, the highest chlorophyll b content (0.63 mg/g) was observed in plants treated with 10 µg/mL of Kn-ZnONPs under 15% PEG-based stress followed by 0.59 mg/g at 10 µg/mL of Kn-ZnONPs at 15% PEG stress, while the lowest content (0.21 mg/g) was observed in plants treated with 100 µg/mL of Kn-ZnONPs grown under 5% PEG. Interestingly, the chlorophyll b content remained non-significant in control-treated plants grown under various levels of PEG-based stress. Moreover, the carotenoid content was also significantly different among various treatments (Kn-ZnONPs and PEG) applied to *V. radiata*. Similarly, when the plants were treated with Kn-ZnONPs at a concentration of 10 µg/mL grown under 10% PEG-induced drought stress, the carotenoid content was 0.09 mg/g. However, increasing the concentration of Kn-ZnONPs to 100 µg/mL resulted in a significant increase in carotenoid content to 0.14 mg/g, regardless of the concentration of PEG-induced drought stress. The highest carotenoid content was observed when the plants were treated with Kn-ZnONPs at a concentration of 200 µg/mL under 10% PEG-induced drought stress, with a carotenoid content of 0.17 mg/g (Figure 11).

Similarly, the results indicated that both Kn-ZnONPs and PEG-induced drought stress had a significant effect on the total chlorophyll content of *V. radiata*. The highest total chlorophyll content was observed at an intermediate level of both treatments, and the content decreased as the concentration of Kn-ZnONPs and PEG-induced drought stress increased. The lowest total chlorophyll content (mg/g) was observed in plants without treatments of Kn-ZnONPs exposed to the highest level (15%) of PEG-induced drought stress, with a value of 0.48 mg/g. The highest value (0.78 mg/g) was observed in treated plants under the same level of PEG-based drought stress with a foliar application of 10 µg/mL of Kn-ZnONPs. Interestingly, the total chlorophyll content of the plants was not significantly affected by the concentration of Kn-ZnONPs when the level of PEG-induced drought stress was either 5% or 15%. Moreover, when the concentration of Kn-ZnONPs was increased to 100 or 200 micrograms/mL, the total chlorophyll content of the plants decreased irrespective of the level of PEG-induced drought stress (Figure 10). The least anthocyanin content of 0.89 mg/g was observed in plants under 5% PEG drought stress without applied Kn-ZnONPs. The highest anthocyanin content was observed in plants treated with 10 µg/mL of Kn-ZnONPs and 15% PEG-induced drought stress, with a value of 2.09 mg/g. When the concentration of Kn-ZnONPs was increased from 10 to 200 µg/mL, the anthocyanin content of the plants showed a slight increase, except in plants subjected to 10% PEG-induced drought stress. These results suggest that the application of Kn-ZnONPs significantly restored the plant photosynthetic pigments that had significantly declined under PEG-based drought stress (Figure 11).

**Figure 12:**
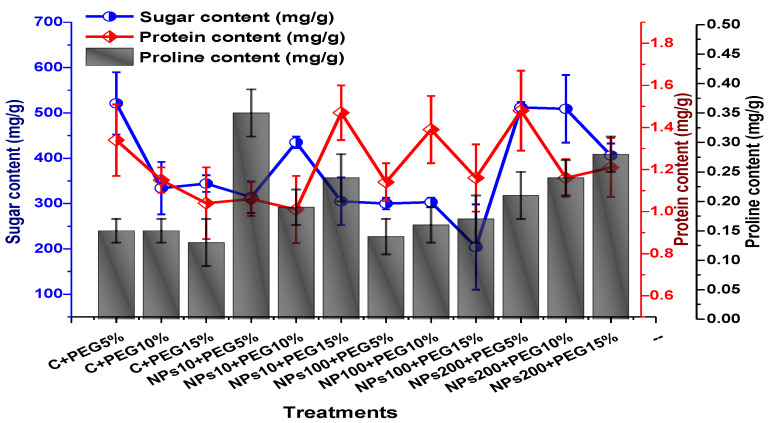
Showing the effect of priming and foliar application of various doses of Kn-ZnONPs on various osmolytes (Sugar, protein, and proline in mg/g FW) of *V. radiata* grown under different levels of PEG-induced drought stress.

ANOVA results revealed a significant variation in soluble sugar content, proline, and protein contents of plants treated by various concentrations of Kn-ZnONPs via the foliar route and grown under PEG-based drought stress. An increase in soluble content (up to 521 mg/g) in leaves was observed at 5% PEG-induced drought stress, whereas a decline in sugar content (up to 204 mg/g) was reported in plants treated with 100 µg/mL Kn-ZnONPs under 15% PEG-based drought stress. ANOVA revealed maximal protein content in plants exposed to 5% PEG-based stress and treated with 200 µg/mL, while minimal protein content was recorded in 10 µg/mL of NPs-treated plants under 10% PEG drought stress. High drought stress (15% PEG) inhibited proline content, whereas maximum proline contents were recorded (0.35 mg/g) in plants treated with10 µg/mL of Kn-ZnONPs under 5% PEG drought (Figure 12).

### 2.4. Effect of Kn-ZnONPs on Antioxidant Biosystem

**Figure 13:**
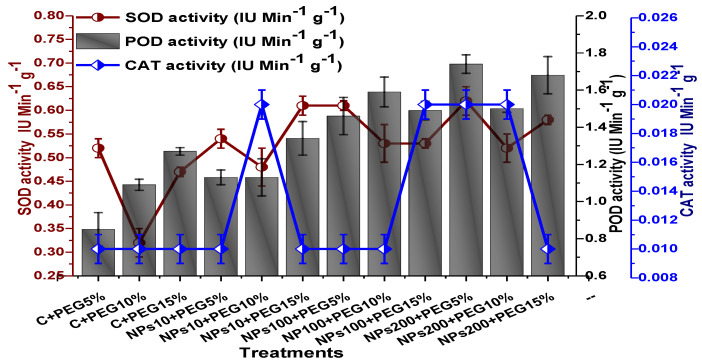
Showing the effect of priming and foliar application of various doses of Kn-ZnONPs on the activity of antioxidant enzymes (SOD, POD, and CAT in IU min^−^^1^ g^−^^1^) of *V. radiata* grown under different levels of PEG-induced drought stress.

**Figure 14:**
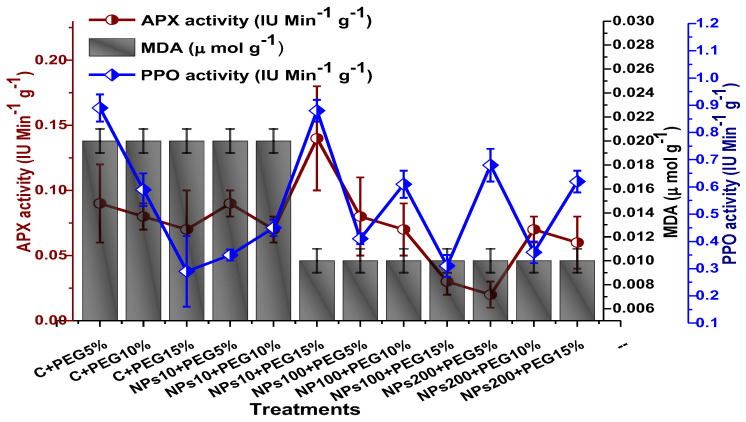
Showing the effect of priming and foliar application of various doses of Kn-ZnONPs on the activity of antioxidant enzymes (APX, PPO IU min^−^^1^ g^−^^1^ and MDA in µmol g^−^^1^) of *V. radiata* grown under different levels of PEG-induced drought stress.

**Figure 15:**
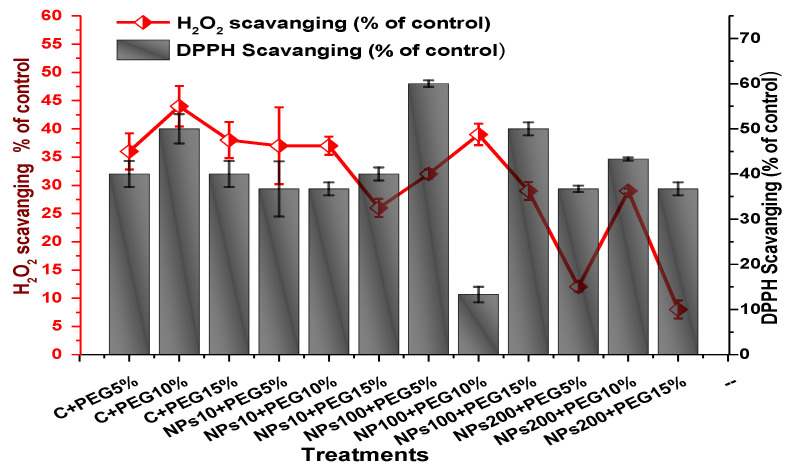
Showing the effect of priming and foliar application of various doses of Kn-ZnONPs on free radical scavenging (H_2_O_2_ and DPPH) potential of *V. radiata* grown under different levels of PEG-induced drought stress.

**Figure 16:**
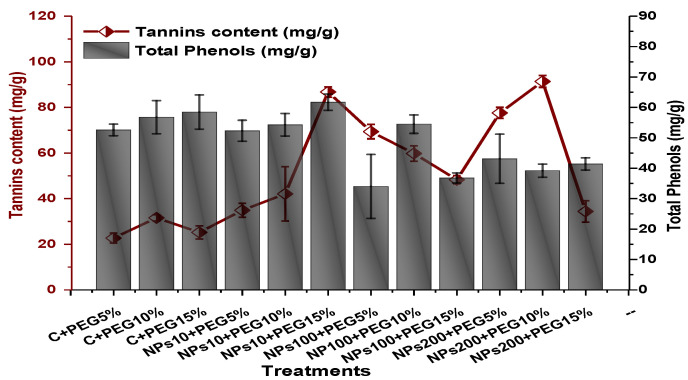
Showing the effect of priming and foliar application of various doses of Kn-ZnONPs on total phenolic and tannin content of *V. radiata* grown under different levels of PEG-induced drought stress.

The impact of Kn-ZnONPs on the antioxidant enzymes superoxide dismutase (SOD), catalase (CAT), ascorbate peroxidase (APX), and peroxidase (POD) of *V. radiata* grown under PEG-induced drought stress was also evaluated. PEG-based drought stress significantly affected the SOD activity and showed minimal activity (0.32 IU/min/g FW) at 10% PEG stress level followed by (0.47 IU/min/g FW) at 15% PEG stress level in plants that remained untreated by Kn-ZnONPs, whereas maximal SOD activity (0.61 IU/min/g FW) was recorded in plants treated with 100 µg/mL of Kn-ZnONPs grown under 5% PEG-based drought stress (Figure 13). Interestingly, POD activity was enhanced by increasing the level of drought stress, where minimal activity was recorded at 5% PEG drought stress and its highest activity was observed in plants treated with 200 µg/mL of Kn-ZnONPs through foliar spray and grown under 15% PEG drought stress. Likely, the maximal CAT activity was observed (0.02 IU/min/g FW) in plants synergistically treated with 10 µg/mL and 10% of Kn-ZnONPs and PEG, respectively (Figure 13). Plants that received Kn-ZnONPs at a dose of 10 µg/mL and were grown under 15% PEG-based drought stress showed highest APX activity (0.14 IU/min/g FW), whereas its lowest activity (0.02 mg/g) was observed in plants grown under 5% PEG drought stress and treated with 200 µg/mL of Kn-ZnONPs. The results also revealed that hydrogen peroxide scavenging potential was high in plants grown under 10% PEG drought stress without foliar application of Kn-ZnONPs (0 µg/mL), while the lowest value was recorded for plants treated at 200 µg/mL of Kn-ZnONPs under 15% PEG drought stress (Figure 14). Further, MDA (malondialdehyde) concentration or lipid peroxidation activity was observed at a maximum at 100 µg/mL applied NPs under 10% PEG drought stress, while the least lipid peroxidation rate was recorded under 5% PEG and 10% PEG drought stress without NPs treatment. However, high drought stress levels (15% PEG) showed elevated MDA concentrations (0.27 µMol g^−1^ and 0.31 µMol g^−1^) at different NPs concentrations, whereas a maximum decline in MDA concentration was shown by the synergistic effect of NPs at a dose of 200 µg/mL and 15% PEG stress (Figure 14). The antioxidant potential exhibited by the extraction of scavenging DPPH radicals was highest in plants grown under 10% PEG drought stress. The maximal DPPH radical scavenging potential (53.7%) was exhibited by plants under stress, without applied NPs, followed by (50.6%) of plants grown under 15% PEG with a foliar spray of 100 µg/mL of Kn-ZnONPs. Minimum DPPH scavenging activity was observed in plants treated with 100 µg/mL under drought 10% PEG (Figure 15). The increase in PPO activity due to drought stress was declined by the combined impact of Kn-ZnONPs (10 µg/mL, 100 µg/mL, and 200 µg/mL) and PEG drought stress. Findings revealed an increase in total phenolic content (TPC) with increasing drought stress, and this response was also observed for some treatments in tannin content. Plants treated with 10 µg/mL of Kn-ZnONPs under 15% PEG drought stress accumulated maximum phenolic content (61.7 mg/g), and the least TPC (34.0 mg/g) was observed in plants treated with 100 µg/mL and under 5% PEG drought stress. The data for tannin content indicates low tannin content in plants under drought stress, and minimum tannin content (0.02 mg/g) was recorded under 5% PEG without NPs (control) application, whereas NPs treatments enhanced tannin content in the plants (Figure 16). The synergistic effect of Kn-ZnONPs at a dose of 200 µg/mL with 10% PEG increased tannin content (1.11 mg/g) in the plants.

## 3. Discussion

The present study involved the synthesis and characterization of Kn-ZnONPs and their effect on mung bean growth under various levels of PEG-induced drought stress. PEG-induced drought stress significantly decreased all growth parameters, such as morphological traits, physiological traits, and biochemical characteristics, and drastically affected the quality and quantity of their yield [29]. Moreover, we report that the application of various NPs used as nano-fertilizers depicts the most significant effect on plant growth, crop quality, and quantity of yield, and it also attenuates the drastic effect of different abiotic stresses [30].

All growth parameters, such as seed germination, shoot length, root length, shoot, root and leaf fresh and dry weight, number of leaves, and leaf area, were monitored in the present research work. It has been concluded, based on the current study, that all the above growth parameters declined in those plants exposed to various concentrations of PEG-induced drought stress, while on other hand, all these growth attributes increase significantly in mung beans with the application of Kn-ZnONPs. These results are congruent with the results of Ul- Haq et al. [31] who recently reported the significant effect of Glutamic acid capped iron nanoparticles (Glu-FeNPs) on mung bean plants under various concentrations of salinity stress. Mazhar et al. [32] also determined the beneficial effects of iron nanoparticles on the growth parameters of flax plants under water stress conditions. The analysis of the results has revealed that all growth parameters were effectively enhanced. This increase in all growth parameters may be due to the pivotal role of Kn-ZnONPs because the application of Kn-ZnONPs regulates the concentration of kinetin in plants, which enhances cell proliferation, cell elongation, promotes the apical meristems, embryogenesis, triggers callus differentiation [33], and consequently improves plant height, leaf area, root length, number of leaves, and number of pods. Furthermore, it improves the quantity and quality of yield [34] by regulating the work of different enzymes that transport soluble carbohydrates and reduce the contents of nitrates [27]. A lot of the literature data show that ZnONPs play the most potent role in plant growth and development. Additionally, in the present study, we determined that kinetin zinc oxide nanoparticles may also be responsible in the improvement of mung bean growth under various levels of drought stress. As the research data depicted, ZnONPs significantly affect leaf chlorophyll contents, improve sugar and protein contents, and, consequently, improve plant growth and development [35]. Various pathways have been suggested by researchers for nanoparticle association and absorption. It is reported that NPs are mostly absorbed by the stomatal openings of leaves after foliar spray; however, this uptake of NPs is greatly influenced by the NPs size. After entering the stomatal opening, it is transported via the xylem and finally accumulates in the central vacuole.

The survival of life depends upon photosynthesis. The photosynthetic pigment is a very essential factor in the process of photosynthesis and is very important for plant development and growth. To comprehend the status of photosynthesis, the total chlorophyll content was analyzed. In the present study, it was found that chlorophyll contents, such as ch. A, ch. B, carotene contents, and anthocyanin are also badly affected by drought stress. On the other hand, the plants treated with different doses of Kn-ZnONPs showed a significant improvement in chlorophyll contents, indicating that Kn-ZnONPs play synergistic roles in the growth of mung bean plants under various PEG-induced drought stresses. These results show a close resemblance to the research data of [36], studying the response of *Cyamopsis tetragonoloba* L. toward Zinc oxide nanoparticles (ZnONPs), and also with the results of Pandey et al. [37] and Sun et al. [38], who use ZnONPs in 100 µg/mL to study the response of a tomato plant. The beneficial effects of Kn-ZnONPs might be due to the presence of kinetin, which maintains the level of kinetin in a suitable range as the overdose of kinetin shows a drastic effect on plant growth and development [39]. Under drought stress, the degradation of chlorophyll contents acceded due to the amelioration of derogative enzymes, such as chlorophyllase, and consequently impaired biosynthetic pathways [40], while Kinetin had the potential to attenuate the chlorophyllase activity [41]. The application of kinetin improved the uptake of nitrogen, which enhanced the production of rubisco and also regulated the function of the stomata, which maintain the concentration of carbon dioxide and water and thus increase the process of photosynthesis [42]. The zinc provided by the NPs played an effective role in the synthesis of carbonic anhydrase enzymes which aid in the transportation of carbon dioxide in the process of photosynthesis. In corroboration to our results, recently, Ahenger et al. [43] and Nahar et al. [44] demonstrated that the application of kinetin and polyamine enhanced the chlorophyll contents under various stress in different plants, but the combined effect of kinetin and zinc oxide nanoparticles on chlorophyll and photosynthesis has not been reported.

A lot of research data affirm that high levels of proline are better and protect the plants from damages caused by growing under different abiotic stress environments. In this protein, soluble sugar and proline contents were analyzed to ascertain the response of *V. radiata* toward Kn-ZnONPs exposed to different levels of drought stress. Throughout the analysis, it was recorded that all of the above parameters were negatively affected by PEG-induced drought stress and the quantity declined in the plant leaves that were exposed to only drought stress. While these contents were recorded at maximum during the analysis of the research data in plants treated with Kn-ZnONPs, the proline was reported as the most common, important, and common osmolyte which protected cell proteins from denaturation and from the deleterious effects of reactive oxygen species (ROS). The proline also acted as a signaling agent, triggering the expressions of specific genes which contribute to ROS elimination [45]. The increase in the concentration of osmolyte was due to the application of Kn-ZnONPs. It is obvious from the literature that zinc promotes the function of the stomata, and the kinetin provided by Kn-ZnONPs also helps in the elimination of ROS, thus, impeding photo-inhibition and membrane damage. Consequently, biosynthetic pathways are improved, which results in an increased level of sugar and proteins. In corroboration with our results, Ahanger et al. [46] reported that the application of kinetin and spermidine promotes the concentration of osmolytes.

Antioxidant enzymes, such as peroxidase (POD), superoxide dismutase (SOD), and catalase (CAT), are the most active and important components of a plant’s defense system. SOD converted the O_2_- to H_2_O_2_ and this resulted in H_2_O_2_ being catalyzed by POD [47]. Oxidative damage was recorded at a minimum in plants treated with Kn-ZnONPs under PEG-induced drought stress. The results of our research work are in accordance with the results of de Moura et al. [48] who demonstrated that the foliar application of kinetin enhances the antioxidant potential of anthurium and consequently promotes the photosynthesis process. Li et al. [41] and Mehri et al. [49] also reported the ameliorating effect of putrescin, spermidine, and kinetin under cadmium-induced stress, but research discussion on the combined effects of kinetin and zinc NPs are not reported yet. The literature has revealed that the exogenous application of kinetin improves the accumulation of tocopherol, which is an important antioxidant molecule that exists in the chloroplast of plant cells and neutralizes OH- and superoxide. It is also reported that tocopherol enhances stress tolerance ability by improving proline contents [50].

Phenol and tannin are considered the most important secondary metabolites which have an effective role in plant growth and development under various stress environments. In the present work, we found that Kn-ZnONPs show a significant effect on the phenol and tannin content present in the test plants under drought stress (Figure 16). The results of Ahanger et al. [46] show a close resemblance with our results. Phenolic contents, polyamine, ferulic acids, and caffic acid combined and enhanced their stability, while also promoting translocation [51]. The increased accumulation of total phenolic and other secondary metabolites significantly promotes various enzymatic activities which are involved in their biosynthesis. The increased number of secondary metabolites also up-regulate the activities of antioxidant enzymes which protect the cellular machinery and also promote their function [52].

## 4. Materials and Methods

### 4.1. Synthesis of Kn-ZnONPs and Characterization

Kn-ZnONPs were synthesized via co-precipitation method following the protocol of Gnanasangeetha et al. [53]. Aqueous Zn (CH3COO)_2_·2H_2_O (0.02 M) and 2.0 M NaOH were mixed, and pH was adjusted to 12.0 using a NaOH solution. Mixture was kept for 2 h stirring. After completion of precipitation, supernatant was decanted, and precipitates were washed several times with deionized water, centrifuged at 4000 rpm for 10 min, kept for drying in an oven at 100 °C, and then incinerated to 500 °C, resulting in ZnONPs. Aqueous kinetin was mixed in the ZnONPs suspension by vigorous stirring for 48 h, using a magnetic stirrer. The precipitates were washed with deionized water and centrifuged. Supernatant was separated and precipitates were completely dried in a warm air oven at 100 °C. Various complementary analytical techniques (FTIR, XRD, SEM, SEM-EDS, Zeta Potential, and DLS) were employed to characterize the resulting Kn-ZnONPs for its morphology, size, size distribution, crystallinity, and functional groups associated with Kn-ZnONPs at the Department of Pharmacy, CRL (Centralized Resource Laboratory) and MRL (Materials Research Laboratory), University of Peshawar, Pakistan.

### 4.2. Laboratory Experimental Detail

A laboratory experiment was conducted at the Plant Ecology Laboratory, Department of Botany, University of Peshawar, Pakistan. The seeds of *Vigna radiata* were primed with various concentrations of Kn-ZnONPs (hydro-primed, 10 µg/mL, 100 µg/mL, 200 µg/mL) of Kn-ZnONPs and sown in Petri plates under PEG-induced drought stress (5%, 10%, 15%) in a growth chamber at 30 ± 3 °C. Percentage germination (*PG*), mean germination time (*MGT*), germination index (*GI*), mean daily germination (*MDG*), and coefficient of velocity germination (*CVG*) were determined using the following equations:

$$PG=\frac{Gt}{N}\times 100$$

$$MGT=\frac{\Sigma \left(nd\right)}{n}$$

$$GI=\left(7\times G1\right)+\left(6\times G2\right)+\left(5\times G3\right)+\ldots \left(1\times Gn\right)$$

$$MDG=\frac{FGP}{d}$$

$$CVG=\frac{G1+G2+G3+\ldots +Gn}{\left(1\times G1\right)+\left(2\times G2\right)+\ldots +\left(n\times Gn\right)}$$

where:

*Gt* is the total number of seeds germinated, *d* is the number of seeds germinated in a day, *N* is the total number of seeds, while *n*…1 represents the days of germination, *FGP* is the final germination percentage and *G*1–*Gn* represents the number of seeds germinated from day 1 to the last day.

### 4.3. Field Experiment Detail

A field experiment was carried out at the Department of Botany, University of Peshawar, Pakistan (34.0086° N, 71.4878° E) under natural temperature (26–37 °C), light, and humidity conditions (mean 32%). The study employed a completely randomized block design (CRBD) with three replications and ensured the random assignment of all treatments to each experimental unit. Seeds of *V. radiata* were obtained from the National Agriculture Research Center (NARC) in Nowshera, Pakistan and sterilized with 70% ethanol. Sand loamy soil was filled in pots, and seeds were sown, followed by growth of seedlings under normal day–night conditions. Young plants were treated with different doses of Kn-ZnONPs (10 µg/mL, 100 µg/mL, 200 µg/mL) through foliar spray at 4-day intervals, while various levels of drought stress were induced through foliar spray of different concentrations of PEG (5%, 10%, 15%). Vegetative growth parameters, including root and shoot length, leaf fresh and dry weight, root fresh and dry weight, number of leaves, and leaf area, were measured for fresh plants. The harvested plants were packed in zipper bags and stored at −20 °C for determining physiological and biochemical parameters.

### 4.4. Measurement of Biochemical Parameters

#### 4.4.1. Measurement of Proline Protein and Soluble Sugar Contents

To determine the proline content, leaves weighing 0.5 g were homogenized in sulfosalicylic acid and then centrifuged for 10 min at 4000 rpm. Then, 0.1 mL of the resulting supernatant was mixed with 2 mL of acidic ninhydrin in a test tube and incubated in a water bath at 100 °C for one hour, followed by cooling in an ice bath. After that, 4 mL of toluene was added to the test tube, and the optical density of the resulting mixture was measured at 520 nm using toluene as a blank.

The protein content was determined using 0.5 g of fresh leaves, homogenized in 1 mL of phosphate buffer (pH 7.5), and the total protein content was determined using the method described by Rostami and Ehsanpour [54].

For the determination of soluble sugar content, 0.5 g of fresh and healthy mung bean leaves were homogenized in 10 mL of distilled water and then centrifuged for 15 min. Next, 0.1 mL of the supernatant was mixed with 5 mL of concentrated H_2_SO_4_ and 80% phenol, and the optical density (OD) of the resulting reaction mixture was measured at 420 nm using a UV-Vis spectrophotometer according to the method described by Dubois et al. [55].

#### 4.4.2. Measurement of Photosynthetic Pigments

To quantify the photosynthetic contents, 0.5 g of leaves were homogenized in 4 mL of 80% acetone. The resulting mixture was then kept in the dark for two hours, followed by centrifugation. The optical density (*OD*) was recorded at wavelengths of 645 nm, 663 nm, and 470 nm. The values obtained were then used to calculate the photosynthetic pigments as described by Gholami et al. [56] using the appropriate equations as

$$Chl\;a\left(mg/g\right)=\frac{\left(12.25\times OD\;at\;663-2.79\times OD\;at\;645\right)\times V}{1000\times LW}$$

$$Chl\;b\left(mg/g\right)=\frac{\left(21.5\times OD\;at\;645-5.1\times OD\;at\;663\right)\times V}{1000\times LW}$$

$$Carotenoid\left(mg/g\right)=\frac{\left(1000\times OD\;at\;663-2.79\times OD\;at\;645\right)\times V}{1000\times LW}$$

#### 4.4.3. Measurement of Antioxidant Enzymes (SOD, POD, APX, and CAT)

The quantification of antioxidant enzymes involved homogenizing 0.5 g of fresh leaves in 0.05N PBS (pH 7.0) containing PVPP and 0.1 M EDTA. For evaluating the potential of peroxidase (POD, EC 1.11.1.7), 0.1 mL of enzyme extract supernatant was mixed with 0.1 mL of phenylene diamine, 100 mM of MES buffer, and 0.05% H_2_O_2_. The change in OD was recorded at 485 nm for 3 min, and the potential of POD was presented as delta OD 485 nm/min mg protein. To determine the potential of superoxide dismutase (SOD, EC 1.15.1.1), 0.1 mL of enzyme extract in 50 mM PBS (pH 7.8) was added to 0.1 mM EDTA, 0.075 mM NBT, 13 mM methionine, and 0.002 M riboflavin and placed below the light chamber for 10 min, and the OD was recorded at 560 nm [57]. The activity of catalase (CAT, EC 1.11.1.6) enzyme was determined according to the protocol of Aebi et al. [58]. Fresh leaves of 1.5 g were homogenized in Tris-HCL of 100 mM containing Dithiothreitol of 5 mM, MgCl_2_ 10 mM, EDTA 1 mM, magnesium acetate 5 mM, PVP-40 of 1.6%, aphenylmethanesulfonyl fluoride of 1 mM, and aproptinin 1 µg mL^−1^, mixed and centrifuged for 10 min at 8000 rpm, and the supernatant OD was recorded at 470 nm. APX potential was recorded following the protocol of Saxena et al. [59].

#### 4.4.4. Measurement of Lipid Peroxidation and PPO

To determine the rate of lipid peroxidation, 600 mg fresh leaves of the sample were grounded in 0.1% TCA and centrifuged for 10 min at 8000 rpm. Then the supernatant was added to thiobarbituric acid of 0.5% and TCA, heated for 35 min at 92 °C, cooled and centrifuged again at 8000 rpm. The OD of the reaction mixture was recorded at 532 nm. PPO potential was recorded following the protocol Alhakmani et al. [60].

#### 4.4.5. Measurement of Total Phenolic and Tannin Contents

Total phenolic contents (TPC) were determined by following the procedure of Ul-Haq et al. [15], with slight modification. About 20 µL extract of leaves, 90 µL of folin-ciocalteu reagent, and 90 µL of Na_2_CO_3_ were mixed and the OD of the reaction mixture was recorded at 630 nm. The tannin content in the leaves of the test plant was evaluated following the method of Chen and Zhang [61].

#### 4.4.6. Measurement of Hydrogen Peroxide (H_2_O_2_) and DPPH Scavenging Activity

To determine the antioxidant potential of plant specimens under applied Kn-ZnONPs, 20 µL of fresh leaves sample and 180 µL of DPPH solution in methanol were combined in a 96-well microplate. The reaction mixture was placed for one h in the dark and the OD was determined at 517 nm following the protocol of Singleton and Rossi [62]. The hydrogen peroxide (H_2_O_2_) scavenging potential was evaluated using the method of Velikova et al. [63], followed by [64,65,66]. For this purpose, 0.1 g of fresh mung bean leaves were homogenized in 0.1% TCA and centrifuged for 15 min at 8000 rpm. A test sample of 0.5 mL was mixed with H_2_O_2_ in a phosphate buffer in a test tube to which 1.0 M of KI was added and the OD was measured at 390 nm with the help of a spectrophotometer.

## 5. Conclusions

*V. radiata* showed distinctive responses for germination, growth parameters, assimilatory products, and antioxidant enzyme activities when evaluated in light of the applied Kn-ZnONPs treatments and PEG-induced drought stress. Furthermore, the application of Kn-ZnONPs to the plant under drought stress showed a positive effect in terms of its growth, photosynthetic pigments, sugar content, protein content, and proline content. Moreover, drought stress at various levels enhanced the activity of antioxidant enzymes, including SOD, POD, APX, and CAT, whereas the reduced activity of PPO and DPPH was observed. Thus, the application of Kn-ZnONPs has been shown to minimize the negative effects of PEG-induced drought stress by ameliorating drought stress resistance in the plant.

## Data Availability

Not applicable.
